# Commentary: Is There an “Acquired Idiopathic Head-Shaking Nystagmus?”: A Discussion of Mechanisms and Clinical Implications Based on a Case Report

**DOI:** 10.3389/fneur.2022.955081

**Published:** 2022-06-22

**Authors:** Sun-Uk Lee, Ji-Soo Kim

**Affiliations:** ^1^Department of Neurology, Korea University Medical Center, Seoul, South Korea; ^2^Department of Neurology, Seoul National University College of Medicine, Seoul, South Korea; ^3^Department of Neurology, Dizziness Center, Clinical Neuroscience Center, Seoul National University Bundang Hospital, Seongnam, South Korea

**Keywords:** vertigo, nystagmus, benign recurrent vertigo, head-shaking nystagmus, dizziness

We read the article by Filippopulos and his colleagues with great interest (1). This report described a patient with recurrent episodes of vertigo prompted by fast head movement (1). The vertigo was accompanied by a severe right-beating head-shaking nystagmus (HSN) with an increased time constant of 60 s. Despite scrutinized neurotologic and radiologic evaluation, there was no evidence of peripheral or central vestibulopathy, except prominent HSN. The patient had no prior history or subsequent development of any associated symptoms during the 6 years of follow-up, which may exclude the diagnosis of vestibular migraine, Meniere's disease, or vestibular paroxysmia. The authors insisted that this patient represents a distinct clinical entity with an unstable velocity storage mechanism (VSM), thereby assigning a new disorder termed “acquired idiopathic head-shaking nystagmus” (aiHSN).

As the authors noted, we previously reported a group of patients with recurrent spontaneous vertigo with interictal HSN (BRV-HSN) in the absence of peripheral vestibulopathy as was documented by caloric, head-impulse, or rotatory chair tests. Even though we could not assess HSN in those patients with BRV-HSN during the attacks, the only difference between the BRV-HSN and aiHSN appears to be the absent HSN between the attacks in aiHSN. Given the (1) increased time constants of the HSN and rotatory nystagmus, (2) emergence of the HSN even with a short duration of head-shaking, and (3) good responses to baclofen, we proposed that unstable and asymmetric VSM may be the cause for vertigo and BRV-HSN (2). Apparently, the characteristics of HSN observed in the patient reported by Filippopulos et al. (1) do not seem to differ from those observed in our patients with BRV-HSN. Thus, the BRV-HSN and aiHSN may lie on the same spectral line due to a common pathomechanism (unstable and asymmetric VSM), rather than distinct disease entities. In addition, the clinical presentation does not escape from the umbrella term of BRV.

## Author Contributions

S-UL analyzed and interpreted the data and wrote the manuscript. J-SK designed and conceptualized the study, interpreted the data, and revised the manuscript. Both authors contributed to the article and approved the submitted version.

## Funding

This study was supported by the Korea Health Technology R&D Project through the Korea Health Industry Development Institute (KHIDI), funded by the Ministry of Health and Welfare (HI14C3477), and the Basic Science Research Program through the National Research Foundation of Korea (NRF) funded by the Ministry of Education, Science and Technology (no. NRF-2016R1D1A1B04935568).

## Conflict of Interest

J-SK serves as an Associate Editor of Frontiers in Neuro-Otology and on the editorial boards of the Journal of Clinical Neurology, Frontiers in Neuro-Ophthalmology, Journal of Neuro-Ophthalmology, Journal of Vestibular Research, Medicine, and Clinical and Translational Neuroscience. The remaining author declares that the research was conducted in the absence of any commercial or financial relationships that could be construed as a potential conflict of interest.

## Publisher's Note

All claims expressed in this article are solely those of the authors and do not necessarily represent those of their affiliated organizations, or those of the publisher, the editors and the reviewers. Any product that may be evaluated in this article, or claim that may be made by its manufacturer, is not guaranteed or endorsed by the publisher.

## References

[1] Filippopulos F Zwergal A Huppert D. Is there an “acquired idiopathic head-shaking nystagmus”?-A discussion of mechanisms and clinical implications based on a case report. Front Neurol. (2022) 13:897012. 10.3389/fneur.2022.897012 35669878 PMC9163310

[2] Lee SU Choi JY Kim HJ Kim JS. Recurrent spontaneous vertigo with interictal headshaking nystagmus. Neurology. (2018) 90:e2135–45. 10.1212/WNL.0000000000005689 29792303

